# Genome-wide scan for signatures of selection in Hanwoo and Angus cattle using whole-genome sequence data

**DOI:** 10.1371/journal.pone.0324034

**Published:** 2025-05-27

**Authors:** Hyoun Ju Kim, Nasir Moghaddar, Sam Clark, Julius H. J. van der Werf, Sara de las Heras-Saldana

**Affiliations:** 1 School of Environmental and Rural Science, University of New England, Armidale, New South Wales, Australia; 2 AGBU, a Joint Venture of NSW Department of Primary Industries and Regional Development and University of New England, Armidale, New South Wales, Australia; Banaras Hindu University, INDIA

## Abstract

This study used whole-genome sequence data on 406 beef cattle (203 Hanwoo and 203 Angus) to detect signatures of selection using four different methods; integrated haplotype score (iHS), Rsb, XP-EHH, and runs of homozygosity (ROH). Based on Rsb and XP-EHH analysis, 36 and 21 genomic regions differed significantly between Angus and Hanwoo breeds. Within breeds, we identified 108 regions (76 in Hanwoo and 32 in Angus) with the ROH analysis and 331 regions with the iHS method (298 in Hanwoo and 33 in Angus). The candidate genes related to meat quality, such as *HSPA9* and *LPL*, were found within Hanwoo, while genes associated with growth and meat quantity traits, including *ACTC1* and *TMEM68*, were identified within Angus. This study can assist in understanding the selection history of these breeds and identifying the genomic regions associated with the traits selected for in the breeding programs for these cattle breeds.

## Introduction

For several decades, beef cattle have been selected to improve their genetic merit for economically important traits using only phenotypic information and pedigree. Advancements in next-generation sequencing technologies have made it possible and affordable to obtain genomic sequences or higher marker genotypes for many animals, thereby facilitating the identification of genomic regions associated with phenotypic variation in traits, or genomic regions that have been shaped by the history of natural and artificial selection [1,2]. Regions in the genome that have been selected and preserve specific DNA sequences remaining from ancestors under selection are referred to as “signatures of selection” (SoS) [3]. Studying the selection signature within and between breeds provides the opportunity to find genomic

regions and genes related to economic traits that have been selected for and to better understand these populations’ history and structure [1,3–5]. Several methods have been applied to detect the signature of selection. One notable approach is the extended haplotype homozygosity (EHH) method, which involves evaluating the extended linkage disequilibrium of high-frequency haplotypes. This method can detect partial selection sweeps within a population [6], and complete selection sweeps between populations [7,8]. Runs of homozygosity (ROH) describe the reduced local variability related to the given genome coverage [9], interpreted as regions undergoing selection around a target locus [10]. Moreover, ROH is also used to estimate the inbreeding coefficient levels, which helps to describe the population structure and diversity [11]. Several genomic regions can overlap between approaches if strong selection events have occurred [10], but diverse methods of detection also often result in different regions being detected. Therefore, comparing and combining the different approaches is an effective way to detect signatures of selection [12]. Many studies into signatures of selection in beef cattle have been published [13–23]. SoS studies with beef cattle, such as Angus and Brahman, using sample sizes ranging from 31 to 3122 animals and employing high-density genetic markers or whole genome sequence data, have identified genomic regions that contain candidate genes for body size and body structure [12–14] and meat quality [14–16] on *Bos taurus* autosome (BTA) 2, 3, 4, 14, 15 and 16. For Hanwoo, significant regions from such studies were shown to have candidate genes related to the immune system [17–19] and intramuscular fat [18–21] on BTA 2, 3, 9, 10, 11, 12, 22 and 26. Strucken *et al*. [22] found selection signatures by comparing genomic data from Hanwoo and Australian Wagyu cattle with the Fst method, using a 777K single-nucleotide polymorphism (SNP) chip. The study found six candidate genes related to fat metabolism and blood vessel pathway located on BTA 2, 7, 17 and 18 in Hanwoo and other three candidate genes on BTA1 and BTA16 in Wagyu. This study compared two cattle breeds with distinct characteristics. Hanwoo is managed under a national breeding program in Korea, whereas Angus is a widely distributed global breed. Hanwoo is primarily selected for superior meat quality [23,24], while Angus exhibits greater meat yield and larger body size than Hanwoo [25]. These fundamental differences in breeding strategies and selection objectives may contribute to detecting a greater number of genomic regions under selection signatures [26]. The power of a selection signature study, which is the probability of a true effect exceeding the significant threshold, depends on factors such as the SNP density and the methods used. High-density SNP chips or sequencing data can increase the power of detecting signature of selection by using markers in stronger linkage disequilibrium [27]. Applying methods based on haplotype analysis is generally more powerful to identify ongoing selection [27,28]. In addition, Ben-Jemaa *et al*. [29] reported a greater number of signatures of selection when employing the EHH method as differed to using allele frequency in African cattle. Similarly, Liu *et al*. [30] detected a larger number of genomic regions and candidate genes through the EHH method than with ROH analysis in Shanghai Holstein cattle. In this study, we hypothesise that using the whole-genome sequence data for the detection of signatures of selection within and between beef cattle breeds that are distinctly different in phenotype, will enable the identification of genomic regions under strong selection. Therefore, the aim of this study was to detect the signature of positive selection in Hanwoo and Angus cattle using whole genome sequence data and four different detection approaches and identify candidate genes that can be involved in production traits for which these beef cattle breeds were selected.

## Materials and methods

### Ethics statement

The whole genome sequences used in this study for Hanwoo and Angus were collected and reported in previous research [31,32]. Therefore, ethical approval from a committee was not required for the current study.

A total of 406 animals with whole-genome sequence data from two breeds (203 Hanwoo and 203 Angus) were used in this study. Sequences for the Angus breed were obtained from the Run9 of the 1000 bull genome project [32] and Hanwoo were available from Nawaz *et al*. [31]. To ensure balanced dataset for comparative analysis, 203 Angus were selected out of a larger number of whole-genome sequences available. A genomic relationship matrix (GRM) was built via GCTA v1.94.2 software [33] using the SNPs was only utilized exclusively for the Angus breed. A quality control assessment of the genotypes on the autosome was performed in PLINK v1.9 [34] to remove the SNPs with a genotype call rate of less than 95% less than 1% minor allele frequency (MAF), and those with a p-value <0.00001 for Hardy Weinberg equilibrium (HWE). After the quality control, we used 7,868,872 SNPs that overlapped between Hanwoo and Angus sequence data sets. Two SNP densities, 50K SNP arrays and whole-genome sequencing (WGS), were utilized for genomic analyses. In general, 50K SNP data provided sufficient resolution to distinguish population differences between Angus and Hanwoo using principal component analysis (PCA), effective population size or LD-based Ne calculations. However, WGS data was essential for detecting rare variants and characterizing fine-scale population structures with higher precision.

### Genetic structure of the populations

To understand the population structure between and within Angus and Hanwoo cattle, a principal component analysis (PCA) in GCTA v1.94.2 [33] was used based on a GRM derived from the 50K Illumina BovineSNP50 BeadChip. The result of the PCA was visualized using the R package ‘ggplot2’v3.5.1 [35].

### Effective population size (Ne)

The effective population size (Ne) serves as an important parameter to assess the genetic diversity in a population. This play a role in estimating the amount of genetic drift, which influences the evidence that is used to infer genomic changes from processes such as selection, mutation and migration. In this study, Ne was estimated for both Angus and Hanwoo breeds in PLINK v 1.9 [34] using a SNP array for the Illumina BovineSNP50 BeadChip, as follows:

$$N_{e_{t}}=\frac{1}{4_{c}}(\frac{1}{r^{2}}-1),$$ (1)

where *t* is the number of generation ago, *c* is the distance in Morgan between SNPs and is assumed to be 100 Mb per Morgan, and ${\text{r}}^{2}$ is the observed average LD at a given genomic distance c [36].

### Identification of selection signatures within breed

#### Runs of homozygosity (ROH).

Runs of homozygosity (ROH) are the estimated length of segments of continuous homozygosity along a segment of a chromosome, facilitating comparisons both within and between populations. We estimated the ROH with PLINK v1.9 using the following parameters: a sliding window of 50 SNPs, 300 kb as the minimum length of ROH, at least 50 consecutive SNPs, a maximum gap between consecutive SNPs of 1000 kb, a proportion of homozygous overlapping windows of 0.05, and a density of one SNP per 50 kb. The ROH were classified depending on their length into five classes: 0-2Mb, 2-4 Mb, 4-8 Mb, 8-16 Mb, and >16 Mb [37–39]. The genomic inbreeding for each animal was calculated based on the ROH using PLINK v1.9 software and following the equation [40]:

$$F_{roh}=\sum L_{roh}/L_{auto},$$ (2)

where $\sum L_{roh}$ is the sum of all individual’s ROHs and *L*_*auto*_ is the length of the autosomal genome. Results were analysed to calculate the frequency of SNPs in ROH island and visualised with the R package ‘detectRUNS’v 0.9.6 [41].

#### Integrated haplotype score (iHS).

Extended haplotype homozygosity (EHH) is the probability of a segment of a chromosome carrying a haplotype homozygous for an extended region [6]. The genotypes from both breeds were phased to identify haplotypes using Eagle v.2.4 [42]. The EHH was calculated with the R package ‘rehh’ v 3.2.2 [43] following the equation proposed by Sabeti *et al*. [6]:

$$EHH_{s,t}^{a}=\frac{1}{n_{a}(n_{a}-1)}\sum_{k=1}^{k_{s,t}^{a}}n_{k}(n_{k}-1),$$ (3)

where *n*_*a*_ is the number of haplotypes that carry the core allele *a*, which is *a* a focal and the designated variant at marker, $k_{s,t}^{a}$ is the number of haplotypes between focal marker *s* to SNP *t*, *n*_*k*_ is the number of ${\text{k}}^{\text{th}}$ haplotypes. To estimate the integrated haplotype score (iHS) within population, an integrated EHH (iHH) index was calculated as the area under the EHH curve for the core allele *a* within a chromosome [7]. An EHH curve is typically defined as linearly interpolating between consecutive markers, although for sequence data, a stepwise constant function would be more appropriate. The integral is computed with the trapezoidal rule, and these iHH values were then used to estimate an unstandardized iHS (uniHS) according to Voight *et al*. [7]:

$$uniHS=\ln\frac{iHH_{A}}{iHH_{D}},$$ (4)

where *iHH*_*A*_ is the integrated EHH decay of ancestral alleles, *iHH*_*D*_ is the integrated EHH decay of derived alleles. Finally, we calculated standardized iHS values, with a mean of 0 and a standard deviation of 1 for the allele frequency at the core SNP as:

$$iHS=\frac{uniHS-mean(uniHS)}{sd(uniHS)},$$ (5)

### Identification of selection signatures between breeds

For the between population comparison, the site-specific extended haplotype homozygosity (EHHS) was used to calculate both XP-EHH and Rsb, with R package ‘rehh’ v 3.2.2 [43] based on [8]:

$$EHH_{s}s,t=\frac{1}{n_{s}(n_{s}-1)}sum_{k=1}^{k_{s,t}^{a}}n_{k}(n_{k}-1),$$ (6)

where *n*_*s*_ is the number of haplotypes at marker *s*, *k*_*s*,*t*_ is the number of haplotypes between core SNP *s* to SNP *t*, *n*_*k*_ is the number of ${\text{k}}^{\text{th}}$ haplotypes. The integrated un-normalized EHHS (iES) value refers to the area under the EHHS curve at *a* core allele within the chromosome [7].

#### XP-EHH.

For unXP-EHH, the iES value [8] for the Hanwoo (population 1) and Angus (population 2) were used as follows:

$$unXP-EHH=\ln\frac{iES_{pop1}}{iES_{pop2}},$$ (7)

#### Rsb.

The difference between XP-EHH and Rsb is that XP-EHH uses the un-normalized EHHS (iES), while Rsb uses the normalized EHHS value (inES) according to Tang *et al*. [44]:

$$unRsb=\ln\frac{inES_{pop1}}{inES_{pop2}},$$ (8)

The values of unXP-EHH and unRsb were standardized to a standard normal distribution with mean 0 and standard deviation 1 and for each value we then calculated a p-value from the Gaussian cumulative distribution function ϕX as $pX=-\log_{10}\left(1-2|\phi X-0.5|\right)$, X is the iHS, XP-EHH or Rsb.

### Candidate genes, Gene Ontology (GO) and pathway

Regions with a positive signature of selection were identified based on the threshold for significance. Since each method has different data distribution characteristics, specific significance thresholds were applied, such as: the uppermost 0.1% level for ROH; $-\log_{10}\left(p-value\right)>4(p-value<0.0001)$ for iHS; and a 0.05 false discovery rate (FDR) value for XP-EHH and Rsb. We used the Ensembl database for the *Bos taurus* ARS-UCD 1.2 reference genome to identify candidate genes located within the 1 Mb window around the significant SNPs identified. Moreover, we performed a gene ontology (GO) enrichment and pathway analysis with the candidate genes using DAVID v6.7 software [45]. The significant threshold used for the GO and pathway analyses was adjusted p-value with Benjamini-Hochberg cut off at <0.05.

## Results

### Genetic structure

**Fig 1:**
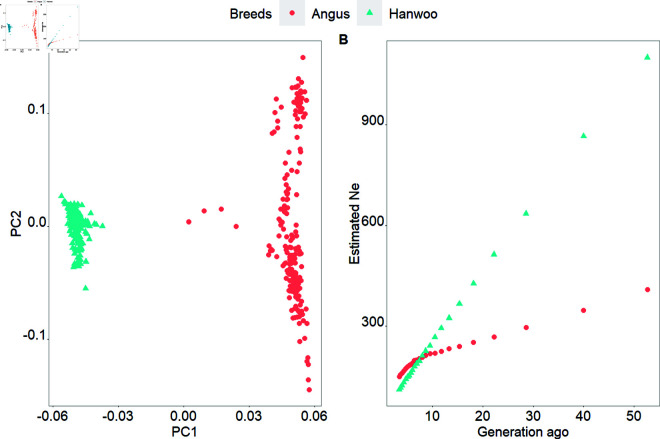
Plot of the first two principal component analysis (A) and the recent effective population size (Ne) trends over time (B) for Hanwoo and Angus breeds.

The PCA plot in Fig 1A showed that Angus and Hanwoo animals were separated in different clusters, and the PC1 and PC2 explained 11.88% and 1.54% of the total variance, respectively. The effective population size (Ne) estimated 5,000 generations ago was higher in Angus (n=4,169) than in Hanwoo (n=4,030) (S1 Fig). However, the Ne in Angus had decreased faster than in Hanwoo, and the level of Ne was higher in Hanwoo until eight generations ago, with each generation in cattle spanning approximately 4 to 5 years. More recently, we estimated the population size three generations ago, Ne is equal to 148 and 110 for Angus and Hanwoo (Fig 1B), respectively.

### Identification of signature of selection in Hanwoo and Angus

**Fig 2:**
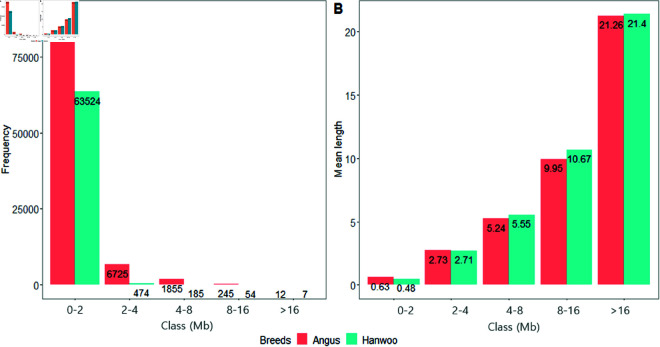
ROH in Angus and Hanwoo. A: by length class frequency. B: by mean length.

In the ROH result, the 0-2 Mb short ROH class was the most frequently observed class in both breeds, with Angus showing a higher frequency (n=89,043) compared to Hanwoo (n=63,524) (Fig 2A). Results also indicated that Angus has higher frequencies than Hanwoo in all ROH classes. On the other hand, the mean length of ROH was longer in Hanwoo than in Angus for classes of 4-8, 8-16 and >16 Mb.

**Fig 3:**
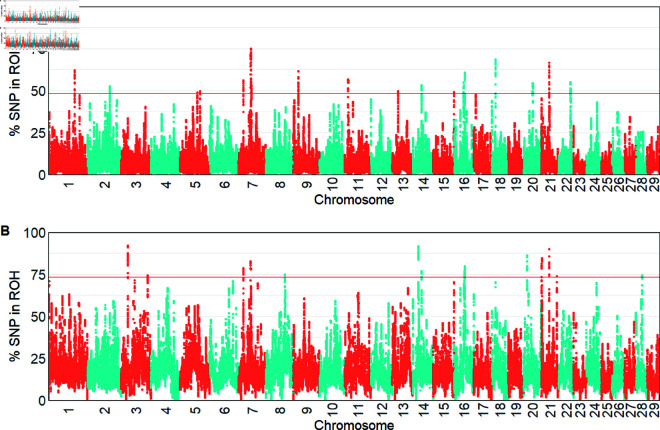
Manhattan plot of percentage of SNPs in ROH across the genome. A: in Hanwoo. B: in Angus.

The selected genomic regions based on the percentage of SNPs in ROH islands were identified across all chromosomes (Fig 3). In total, 76 significant genomic regions and 65 candidate genes were detected in Hanwoo. A larger number of SNP (empirical top 0.1%) in ROH indicates a significant selection signature [46], and the strongest ROH island (74.88%) was located on BTA7 (Fig 3A), with the Heat Shock Protein Family A Member 9 (*HSPA9*) as the closest gene. In Angus, there were 32 significant genomic regions with ROH islands located on BTA 3, 14, 20, and 21, including 30 candidate genes such as transmembrane Protein 68 (*TMEM68*) and LYN proto-oncogene, Src family tyrosine kinase (*LYN*) genes (Fig 3B, S1 Table).

**Fig 4:**
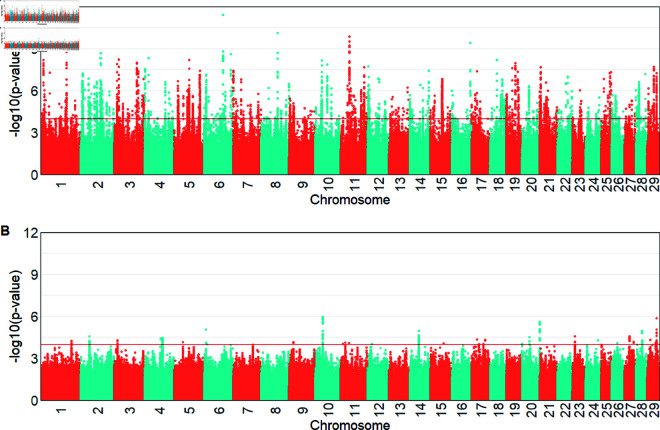
Manhattan plots with significance of iHS values $\left(-\log_{10}\left(p-value\right)\right)$ above the threshold (red line). A: for Hanwoo. B: for Angus.

For the iHS index, from the EHH within the population method, we detected 298 significant genomic regions $\left(-\log_{10}(p-value)>4\right)$ in Hanwoo, expressed as the signature of selection on all chromosomes, with more significant peaks on BTA 2, 6, 8, 11 and 16 (Fig 4A). In Angus, 33 regions and 205 candidate genes were identified as the signature of selection, and there were large peaks on BTA 10, 14, 20, 28 and 29. The most significant iHS values were located on BTA6 (iHS = 6.95) in Hanwoo and BTA10 (iHS = 4.87) in Angus. The candidate genes from within the population in Hanwoo were detected as lipoprotein lipase (*LPL*) and plexin A2 (*PLXNA2*), while the hexokinase 1 (*HK1*), musculin (*MSC*) and actin alpha cardiac muscle 1 (*ACTC1*) were detected in Angus (S1 Table).

**Fig 5:**
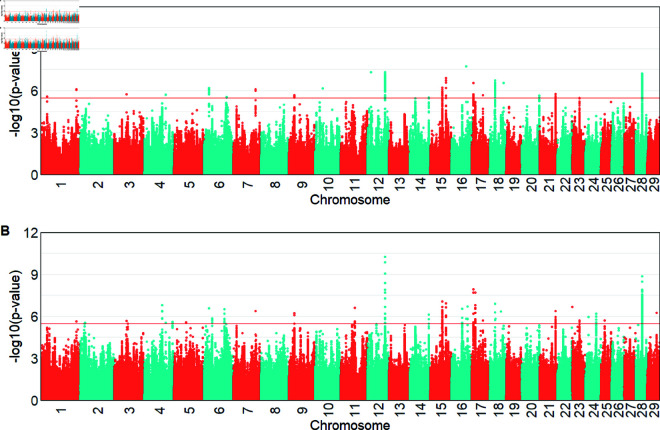
Manhattan plots of the significance values between the populations. A: using XP-EHH. B: using Rsb.

For the between populations analysis, we detected more genomic regions with larger significance values (FDR <0.05) by applying the Rsb method (Fig 5B) compared with using the XP-EHH approach (Fig 5A). The XP-EHH method showed 21 significant regions on BTA 12, 15, 18, 21 and 28, which include 183 candidate genes. The Rsb result revealed 36 significant regions and 371 candidate genes located on BTA 12, 15, 17, 21 and 28. There were five significant peaks found on BTA 12, 15, 21 and 28 that overlapped between methods. The most significant value for Rsb was -6.55 on BTA12, while the XP-EHH approach yielded a -5.63 on BTA16.

The top five significant genomic regions and candidate genes from each approach are shown in Table 1. In the iHS method, we detected nine genes on BTA 1, 6, 8, 11 and 16 for Hanwoo, and 50 genes on BTA 6, 10, 20, 28, and 29 for Angus. All the top five significant genomic regions of ROH were located on BTA7 in Hanwoo, while the regions of ROH in Angus were observed on BTA 3, 14, 20 and 21. In the between breeds analysis, we identified 32 genes using the XP-EHH method and 37 genes with the Rsb method, and the region on BTA29 (24.8-26.1 Mb) overlapped between these two approaches (Table 2).

**Table 1:** Top five most significant genomic regions detected with iHS and ROH methods within breed.

Method	Breed	BTA	Regions (Mb)	Gene acronym
iHS	Hanwoo	1	36.5-37.5	*EPHA3*
		6	75.2-76.2	-
		8	66.1-67.1	*LPL*
		11	34.2-35.2	-
		16	75.2-76.2	*PLXNA2, CD34, CD46, ASPM, ZBTB41, F13B, CRB1*
	Angus	6	7.84-8.87	*TRAM1L1*
		10	29.9-30.9	*GREM1, SCG5, GJD2, ACTC1, AQR, ZN770, DPH6*
		20	70.4-71.5	*IRX4, NDUF56, LPCAT1, SLC6A3, CLPTM1L, TERT, SLC6A19, SLC6A18, NKD2, TRIP13, BRD9, TPPP, CEP72*
		28	24.9-25.9	*TET1, CCAR1, STOX1, DDX50, DDX21, KIFBP, SRGN, VPS26A, SUPV3L1, HKDC1, HK1, TACR2, TSPAN15*
		29	38-39.5	*PAG7, PAG15, PAG4, PAG14, PAG16, PAG20, PAG21, PAG1, PAG19, PAG17, MGC157408, MGC157405, PAG9, PAG3, PAG6, PAG11*
ROH	Hanwoo	7	49.7-49.8	*KDM3B*
		7	49.8-49.8	*REEP2*
		7	49.8-49.9	*ETF1, HSPA9*
		7	50.3-50.4	*SIL1*
		7	50.4-50.5	*SIL1*
	Angus	3	28.3-28.5	*TSPAN2, SYCP1*
		14	23.0-23.0	*TMEM68*
		14	23.2-23.3	*LYN*
		20	14.7-14.8	*SREK1IP1, SHISAL2B*
		21	31.4-31.7	*TMEM266, ETFA, ISL2*

**Table 2:** Top five most significant genomic regions detected with Rsb and XP-EHH methods between breeds.

Method	Breed	BTA	Regions (Mb)	Gene acronym
Rsb	Between-breeds	12	69.9-70.9	-
		15	46.7-49.1	*OR52fam*, *CAVIN3*, *CCKBR*, *CNGA4*, *C15H11orf42*, *HBE2*, *TRIM34*
		17	6.6-7.9	*SH3D19*, *RPS3A*, *LRBA*, *MAB21L2*, *DCLK2*
		17	13.5-14.7	*HHIP*, *GYPA*, *GYPB*, *SMARCA5*, *GAB1*, *USP38*
		28	24.8-26.1	*SLC25A16*, *TET1*, *CCAR1*, *STOX1*, *DDX50*, *DDX21*, *KIFBP*, *SRGN*, *VPS26A*, *SUPV3L1*, *HKDC1*, *HK1*, *TACR2*, *TSPAN15*
XP-EHH	Between-breeds	12	14.6-15.6	*SERP2*, *TSC22D1*, *NUFIP1*, *GPALPP1*, *GTF2F2*, *KCTD4*, *TPT1*, *SLC25A30*, *COG3*
		12	69.9-70.9	-
		15	62.7-63.7	*RCN1*, *WT1*, *EIF3M*, *PRRG4*, *QSER1*, *DEPDC7*, *TCP11L1*, *CSTF3*
		16	59.4-60.4	textitRASAL2, *TEX35*, *RALGPS2*, *ANGPTL1*, *FAM20B*, *TOR3A*
		28	24.8-26.1	*SLC25A16*, *TET1*, *CCAR1*, *STOX1*, *DDX50*, *DDX21*, *KIFBP*, *SRGN*, *VPS26A*, *SUPV3L1*, *HKDC1*, *HK1*, *TACR2*, *TSPAN15*

**Fig 6:**
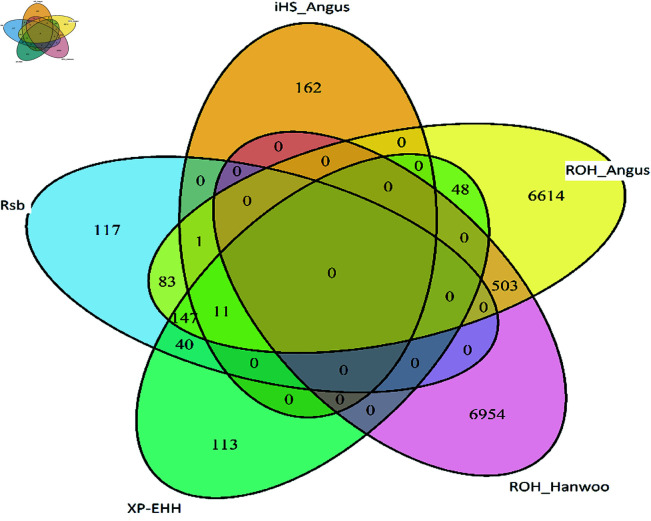
Venn diagram with the number of overlapped significant genomic variants from all methods.

The number of variants overlapping between methods is presented in Fig 6. A larger number of regions were shared between the ROH within Hanwoo and ROH within Angus finding 503 SNPs in common. A total of 147 SNPs overlapped between Rsb and XP-EHH methods within Angus, while 83 shared SNPs were found in common between ROH and Rsb methods within Angus.

### Gene Ontology (GO) and pathway analysis

Candidate genes identified in all methods were used for GO enrichment and pathway analyses with significant results only for candidate genes from the EHH method (S2 Table). The results of GO enrichment analysis among overlapping genes of EHH output are shown in S3 Table. There were five GO terms related to olfactory function with 47 overlapping genes and four GO terms with ten overlapping genes from the result of iHS in Hanwoo. In Angus, two GO terms were significantly enriched in eight GO terms associated with Hemoglobin and oxygen in both XP-EHH and Rsb results.

## Discussion

This study aimed to detect the signatures of selection in Hanwoo and Angus cattle breeds using whole-genome sequence data. Across all methods used, 496 genomic regions were detected from which 36 and 21 significant genomic regions were identified using the Rsb and XP-EHH methods, respectively. A larger number of regions were found from within population analyses, particularly in Hanwoo with 76 and 298 regions, while Angus had 32 and 33 regions when using the ROH and iHS methods, respectively.

### Genetic structure in Angus and Hanwoo

In prior studies, the Ne in Hanwoo three generations ago was estimated to be 98 [47], 327 for four generations ago [48] and 531 for 11 generations ago [45], which differs from our findings of 865 for 40 generations ago, 267 for ten generation ago and 110 for three generations ago, respectively. The differences in the results of Ne between studies could come from the sampling of the animals that were used in the analysis, the number of samples, the relationship between animals that were sampled and the SNP density [49,50]. In the Angus population, the estimated Ne for ten generations ago (Ne = 220) in our study was similar to reported values in a previous study, Ne = 207 [51] and Ne = 64 [52]. In the past, the gap in the Ne level between Angus and Hanwoo might be due to the population size. Angus has a larger population than Hanwoo, and there is no exotic gene flow into the Hanwoo population since it is an isolated population in Korea. Moreover, the effective selection program might have been started earlier in Angus compared to Hanwoo. An organized Hanwoo breeding program started in 1979 under the Ministry of Agriculture and Forestry, but due to insufficient data awareness, it did not yield a significant selection response . Consequently, a new program was initiated in 1999, which provide to be more effective [53]. On the other hand, in the case of Angus, published studies on bull selection date back to the 1970’s [54,55]. Therefore, stronger selection pressure may have been exerted around eight generations ago in Angus, leading to a swifter reduction in the effective population size in Angus compared to Hanwoo.

### Identifying signatures of selection

We used two approaches (ROH and EHH) for detecting signatures of selection in Hanwoo and Angus cattle. Each method uses different statistics focusing either on differences in allele homozygosity within a population (ROH), or haplotype frequencies (EHH). We found that more selection sweeps were revealed in Hanwoo than in Angus from iHS results, while stronger complete selection sweeps were observed in Angus from ROH, XP-EHH and Rsb. In addition, a larger number of variants overlapped between Rsb, XP-EHH and ROH methods in Angus suggesting that Angus could have had a stronger selection history than Hanwoo, as was also confirmed by the ROH and iHS analysis. In the within-population results, regions detected in both populations included 12 regions with 76 genes identified on BTA1, 2, 5, 11, 17, 20, 24, 27, and 29 using the iHS method. In the ROH results, only five common regions with 7 genes were detected on BTA14 and 21. From the analysis of Angus data, BTA16 (42.3-43.8 Mb) and BTA28 (25.3-25.4 Mb) were genomic regions with significant selection signatures detected by the ROH, Rsb, XP-EHH, and iHS methods. The significant genomic region on BTA14 (34.9-35.1 Mb) overlapped between ROH and iHS methods. In Hanwoo, the overlapping regions were located on BTA2 between ROH and iHS. The region on BTA14 (22.7-23.3 Mb), which includes Pleomorphic adenoma gene 1 (*PLAG1*) and Coiled-Coil-Helix-Coiled-Coil-Helix Domain Containing 7 (*CHCHD7*) genes, have been previously identified via ROH and Rsb as a strong selection sweep in both Angus and Hanwoo [13,48], however, this region was not identified in this study. Previous studies mentioned that each selection signature approach could produce different results by applying different statistics [56,57]. The length and number of ROH can reveal some of the breed history [58], with long ROH indicating more recent inbreeding, while the short ROH reflects ancient inbreeding [37]. From the results of ROH in our study, we found Angus has both lengthier and a larger number of ROH, suggesting that Angus has a higher level of inbreeding. For the significant ROH regions, 76 and 32 regions were identified in Hanwoo and Angus, respectively. Although Angus has a higher percentage of SNPs in ROH than Hanwoo, the number of significant ROH regions was smaller in Angus than in Hanwoo because the length of the significant ROH regions was higher in Angus. It also can indicate that Angus has a stronger selection history. This tendency was also observed in the ROH’s mean length and frequency, which was higher in Angus. The mean length and frequency of ROH are evidence of recent selection sweeps. The EHH method serves as a tool for identifying the signature of selection within a population (iHS) and between populations (XP-EHH and Rsb). iHS has proven to be a powerful tool in detecting intermediate selection sweeps as opposed to complete selection signatures [7,9]. Intermediate selection sweeps indicate that certain alleles are under selective pressure, but they are still segregating, rather than completely fixed. A population experiencing weak selection pressure may exhibit a higher number of intermediate allele frequencies in regions that affect a phenotype, compared to a population under strong selection pressure. A higher significance in iHS was found for the Hanwoo breed, suggesting a weaker selection signal than the Angus breed, which had fewer significant iHS results in this study. A stronger selection history for Angus was also revealed by a faster decrease in the effective population size between 3,000 and 7 generations ago for Angus compared to Hanwoo. Since XP-EHH and Rsb are more powerful indices to detect complete selective sweeps [44], the most significant genomic regions from XP-EHH and Rsb identified in Angus breed also showed strong selection evidence from the ROH results. A similar study [59] compared selection signatures between Hanwoo and Angus breeds. Similar to our findings, Nawaz *et al*. [59] described strong selection signals in Angus and highlighted the distinct selection histories, genomic architectures, and breed characteristics. Notably, the same genomic regions were detected in Angus on BTA28, including gene such as DExD-Box Helicase 21 (*DDX21*), Kinesin Family Binding Protein (*KIFBP*), *STGN*, VPS26 Retromer Complex Component A (*VPS26A*), and Hexokinase Domain Containing 1 (*HKDC1*) genes. Similarly, in Hanwoo, selection signatures were observed on BTA17 and 25, encompassing genes such as LPS Responsive Beige-Like Anchor Protein (*LRBA*), Mab-21 Like 2 (*MAB21L2*), and *MS2R* genes. However, there are key methodological differences between the studies related to sample size and the use of whole-genome sequence (WGS) data. While our study utilized WGS data, Nawaz *et al*. [59] relied on imputed WGS data. Additionally, their Angus data originated from the United States, whereas our Angus data were collected from a global dataset [32]. These methodological variations likely contributed to discrepancies in the detected selection regions. Nevertheless, both studies provide valuable insights that can aid in identifying key functional genes associated with the unique genetic characteristics of each breed.

### Candidate genes related to economic traits

In total, 3,437 candidate genes were identified from ROH (81), iHS (2,790), Rsb (383) and XP-EHH (183) methods (S1 Table). The function of these genes and their association with economic traits were investigated and described in the following sections that cover traits related to growth, meat quality, fertility and the immune system. In addition, pathway and gene ontology analyses were performed to identify further biological functions.

#### Growth and meat quantity.

In Hanwoo, the gene dipeptidase 1 (*DPEP1*) was found to be related to feed intake [60], and *MLLT10* has been associated with body stature [61]. In a previous study, Transient Receptor Potential Cation Channel Subfamily V Member 1 (*TRPV1*) has been implicated with growth traits, including body weight, height, and length in three Chinese cattle breeds [62]. From the result of Angus, *TMEM68* and XK-related protein 4 (*XKR4*) genes have been previously associated with feed intake in cattle [14,63,64]. The *XKR4* gene has been reported as related to rump fat thickness in cattle [65]. *ACTC1* is associated with muscle development and fat deposition [61], while Hexokinase Domain Containing 1 (*HKDC1*) is related with glucose metabolism in Angus [66]. In the genome regions that were overlapping in the within and between breed analyses, the gene *HK1* on BTA28 was identified, which was also detected in the result from the within Angus analysis and is related to glucose metabolism within Angus [67]. The *LYN* gene has been associated with body size and stature in Angus [13]. Candidate genes identified in the between-population analyses included the bone morphogenetic protein 7 (*BMP7*) gene, which has been related to bone development and cell growth [60], melanocortin receptor 3 (*MC3R*) previously involved in the body measurement traits and meat quality in Qincian cattle [68], and the Protein phosphatase 1 regulatory subunit 16B (*PPP1R16B*) associated with methane formation in dairy cows [69]. The angiopoietin Like 1 (*ANGPTL1*) gene was detected from the result of XP-EHH, which is critical to anti-angiogenic, and its families play the role of inhibitor of *LPL* [70]. The LPS responsive beige-like anchor protein (*LRBA*) gene was associated with the kinase A, which is related to immune effector molecules [71], and the cholecystokinin B receptor (*CCKBR*) gene was related to the feed efficiency in cattle [72].

#### Meat quality.

Within Hanwoo, we identified the heat shock protein family A member 9 (*HSPA9*) which has been related to tenderness in Chinese cattle [73]. In beef cattle, Prohibitin (*PHB*) is associated with adipocyte differentiation, and the adipogenic (*ADIG*) genes involved in adipogenic differentiation [74]. The *LPL* gene is related to adipose tissue [75], and has been reported to affect the fatty acid composition in Hanwoo [76]. From between populations analysis, the acyl-CoA oxidase 2 (*ACOX2*) gene is associated with lipid storage by breaking down the fatty acid [77], and the pyruvate dehydrogenase E1 subunit beta (*PDHB*) gene is related to intra-muscular fat content in the meat [78,79]. The SWI/SNF related matrix associated actin dependent regulator of chromatin subfamily D member 3 (*SMARCD3*) gene has been implicated in lipid metabolism and muscle cell differentiation [80]. We also detected the cyclin dependent kinase 5 (*CDK5*) gene between populations, and *CDK* families have been found to be related to variation in intra muscular fat [81].

#### Fertility.

In Hanwoo, spermatogenesis Associated 22 (*SPATA22*) plays a role in meiosis [82]. Candidate genes identified in Angus include the neurotrophic receptor tyrosine kinase 2 (*NTRK2*) that has been implicated with the sire conception rate in Holstein cattle [83], and MBL associated serine protease 2 (*MASP2*) was associated with mastitis and milk production in Chinese Holstein cattle [84]. From the results of the between populations analysis, *SPO11* and RAD21 cohesin complex component Like 1 (*RAD21L1*) genes were related to meiosis in Holstein [85], and the SET domain containing 6 (*SETD6*) gene has been detected for fertility in cows [86]. Gonadotropin releasing hormone receptor (*GNRHR*) gene is known to play a role in the age of puberty [87]. Pregnancy-associated glycoprotein (*PAG*) family members were found the result from between populations, which may play a role in the placenta-uterine interface [88].

#### Immune system.

From the between populations result, the candidate genes that were found in this study were previously associated with multiple diseases such as the tetraamelia syndrome in cattle (R-spondin 2 – *RSPO2*) [89], osteopetrosis in Red Angus (Solute carrier family 4 member 2 – *SLC4A2*) [90], liver cancer (MBL associated serine protease 2 – *MASP2*) [84], and mastitis (*BoLA*) [91]. Other genes were associated with resistance to parasites (glycophorin B – *GYPB*) [92], thermotolerance (gamma-aminobutyric acid type B receptor Subunit 1 – *GABBR1*) [93], heat tolerance in Chinese cattle (Mechanistic target of rapamycin kinase – *MTOR*) [94], and cold climate adaptation (mechanistic target of rapamycin kinase – *CORT*) [95]. The significant terms from the gene ontology analysis based on genes identified via iHS in Hanwoo, XP-EHH and Rsb (S2 Table), were related to sensory functions, including olfactory features, smell and taste. The link to olfactory function is important for the general evaluation of animals, as it is essential for survival and communication [96,97]. Similar results have also been reported in previous studies in Hanwoo [97,98], suggesting that Hanwoo is still undergoing selection for survival. Furthermore, olfactory transduction can be associated with feed efficiency in African Sanga cattle [13], and it may influence the growth traits in Hanwoo and Angus. We also found the GO terms oxygen and hemoglobin. The function of hemoglobin is to carry oxygen to muscle, and the consumption of oxygen is related to the meat color in cattle [99,100]. Those GO terms can be associated with the meat quality traits since muscle color is one factor in determining the meat quality grade in Hanwoo. The findings of this study provide a more comprehensive insight into the genomic regions under selection in beef cattle breeds. These results can be leveraged to enhance the efficiency of genetic improvement through their integration into genome-based selection models. Moreover, incorporating previous research on selection signature analysis in Hanwoo enables a comparative study of the selection and diversity regions, which can aid in the preservation of genetic diversity and contribute to long-term breed conservation efforts.

## Conclusion

The aim of this study was to identify the signatures of selection in Hanwoo and Angus breeds using whole-genome sequence data. In Angus, we identified genes associated with growth, meat yield, and fertility, while in Hanwoo, genes were primarily linked to meat quality, fertility, and immune function. The between-population analysis further revealed genes related to both traits, meat quality and quantity, highlighting key regions under selection in each breed. These findings provide insights into the genomic regions influenced by selection for economically important traits and identify SNPs that could serve as predictive markers for genetic differentiation in beef cattle breeding programs.

## Supporting information

S1 Fig The effective population size (Ne) over time for Hanwoo and Angus. (TIF)

S1 Table Candidate genes list from each method. (XLSX)

S2 Table The Gene Ontology (GO) and KEGG pathway of the significant genes for significant genes for signature of selection. (DOCX)

S3 Table The Gene Ontology (GO) enrichment analysis among overlapped genes from all the methods. (DOCX)
