# Supplementary figures and images for: Genome-wide scan for signatures of selection in Hanwoo and Angus cattle using whole-genome sequence data
Source: PLoS One. 2025 May 27;20(5):e0324034. doi: 10.1371/journal.pone.0324034 (PMC12111605; doi:10.1371/journal.pone.0324034)

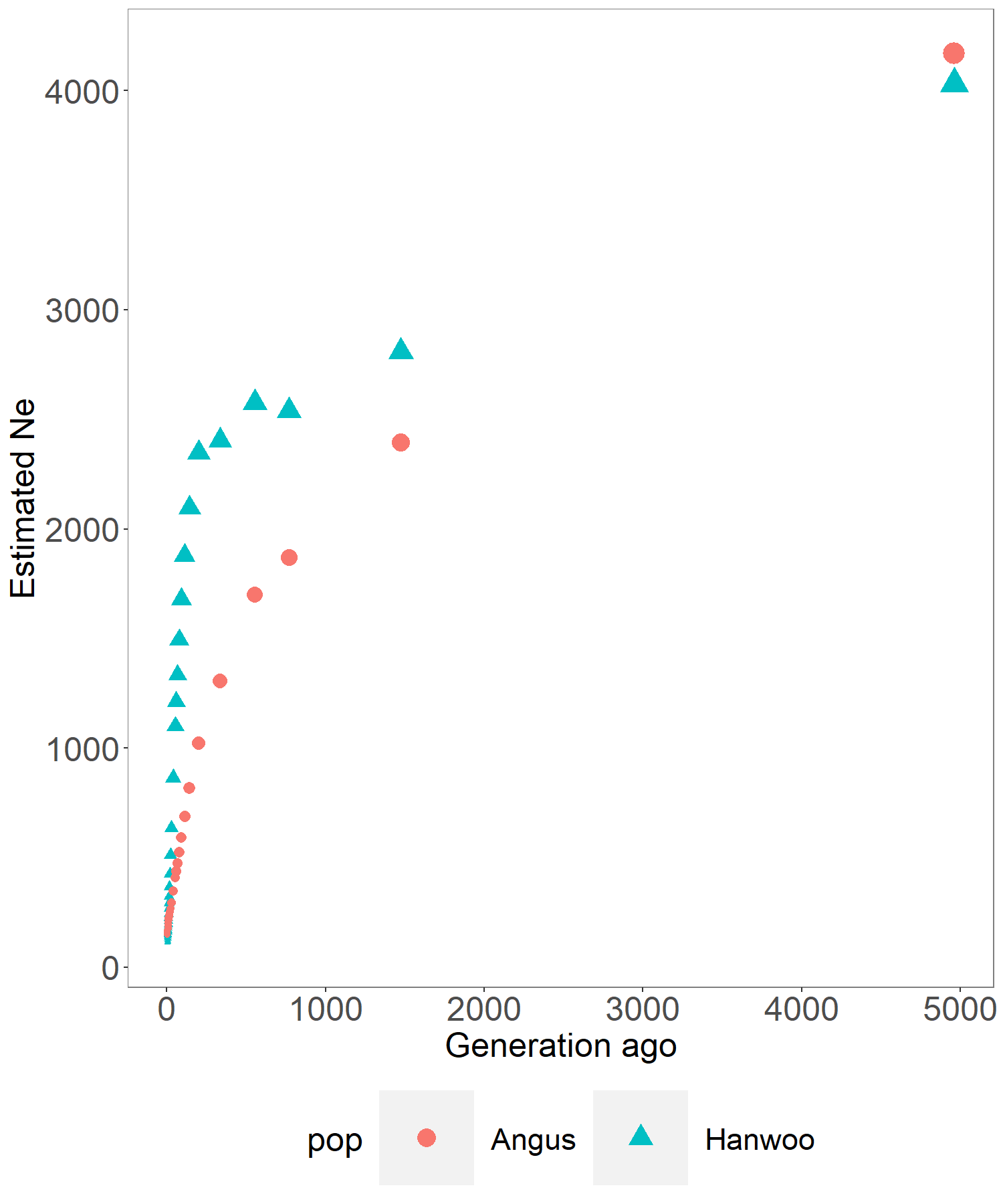

Supplement: S1 Fig — (TIF) [file pone.0324034.s001.tif]
