# Supplementary material for: Genome-wide scan for signatures of selection in Hanwoo and Angus cattle using whole-genome sequence data
Source: PLoS One. 2025 May 27;20(5):e0324034. doi: 10.1371/journal.pone.0324034 (PMC12111605; doi:10.1371/journal.pone.0324034)
Supplement: S2 Table — (DOCX) [file pone.0324034.s003.docx]

**Table 1**. Gene ontology and KEGG pathway analysis of the number of significant candidate genes (n) for signature of selection.

| Methods | Breed | GO term | n | p-value | Benjamini |
| --- | --- | --- | --- | --- | --- |
| EHH(iHS) | Hanwoo | olfactory receptor activity | 362 | 3.80E-97 | 5.20E-94 |
|  |  | G-protein coupled receptor activity | 390 | 8.60E-88 | 5.80E-85 |
|  |  | Olfactory transduction | 355 | 1.40E-56 | 4.80E-54 |
|  |  | odorant binding | 103 | 1.20E-27 | 5.40E-25 |
|  |  | sensory perception of smell | 51 | 3.70E-14 | 1.50E-10 |
|  |  | G-protein coupled receptor signaling pathway | 79 | 2.80E-11 | 5.80E-08 |
|  |  | prolactin receptor binding | 10 | 1.10E-06 | 3.60E-04 |
|  |  | positive regulation of lactation | 11 | 1.90E-06 | 2.60E-03 |
|  |  | detection of chemical stimulus involved in sensory perception of bitter taste | 15 | 2.80E-06 | 2.90E-03 |
|  |  | response to nutrient levels | 11 | 3.80E-06 | 3.10E-03 |
|  |  | CCR chemokine receptor binding | 12 | 4.40E-05 | 1.20E-02 |
|  |  | interleukin-1 receptor binding | 8 | 9.10E-05 | 2.10E-02 |
|  |  | mammary gland development | 12 | 3.40E-05 | 2.40E-02 |
|  |  | detection of chemical stimulus involved in sensory perception of smell | 13 | 6.40E-05 | 3.80E-02 |
|  | Angus | aspartic-type endopeptidase activity | 16 | 1.10E-17 | 2.90E-15 |
|  |  | Protein digestion and absorption | 16 | 2.20E-13 | 3.80E-11 |
| EHH(Rsb) | Between-breeds | olfactory receptor activity | 88 | 3.30E-42 | 8.50E-40 |
|  |  | G-protein coupled receptor activity | 95 | 8.40E-42 | 1.10E-39 |
|  |  | Olfactory transduction | 85 | 3.00E-29 | 6.40E-27 |
|  |  | detection of chemical stimulus involved in sensory perception of smell | 9 | 2.10E-09 | 1.70E-06 |
|  |  | aspartic-type endopeptidase activity | 11 | 4.60E-08 | 4.00E-06 |
|  |  | plasma membrane | 108 | 2.50E-07 | 5.40E-05 |
|  |  | integral component of membrane | 137 | 6.90E-07 | 7.40E-05 |
|  |  | hemoglobin alpha binding | 5 | 1.50E-06 | 9.90E-05 |
|  |  | organic acid binding | 5 | 2.90E-05 | 1.50E-03 |
|  |  | haptoglobin-hemoglobin complex | 5 | 2.20E-05 | 1.60E-03 |
|  |  | hemoglobin complex | 5 | 3.00E-05 | 1.60E-03 |
|  |  | carbonate dehydratase activity | 5 | 9.20E-05 | 3.40E-03 |
|  |  | oxygen transporter activity | 5 | 9.20E-05 | 3.40E-03 |
|  |  | oxygen binding | 5 | 1.80E-04 | 5.90E-03 |
|  |  | hydro-lyase activity | 5 | 2.20E-04 | 6.40E-03 |
|  |  | odorant binding | 14 | 4.30E-04 | 1.10E-02 |
|  |  | oxygen transport | 4 | 4.90E-05 | 2.00E-02 |
|  |  | Nitrogen metabolism | 5 | 3.50E-04 | 3.70E-02 |
|  |  | hydrogen peroxide catabolic process | 5 | 1.70E-04 | 4.60E-02 |
| EHH(XP-EHH) | Between-breeds | hemoglobin alpha binding | 5 | 1.00E-07 | 2.20E-05 |
|  |  | organic acid binding | 5 | 2.00E-06 | 2.20E-04 |
|  |  | haptoglobin-hemoglobin complex | 5 | 2.20E-06 | 2.80E-04 |
|  |  | hemoglobin complex | 5 | 3.10E-06 | 2.80E-04 |
|  |  | oxygen transporter activity | 5 | 6.60E-06 | 4.70E-04 |
|  |  | oxygen binding | 5 | 1.30E-05 | 7.10E-04 |
|  |  | galactoside 2-alpha-L-fucosyltransferase activity | 3 | 1.70E-04 | 5.90E-03 |
|  |  | alpha-(1,2)-fucosyltransferase activity | 3 | 1.70E-04 | 5.90E-03 |
|  |  | CXCR3 chemokine receptor binding | 3 | 3.30E-04 | 9.00E-03 |
|  |  | olfactory receptor activity | 20 | 3.40E-04 | 9.00E-03 |
|  |  | oxygen transport | 4 | 1.80E-05 | 9.80E-03 |
|  |  | hydrogen peroxide catabolic process | 5 | 4.70E-05 | 1.30E-02 |
|  |  | G-protein coupled receptor activity | 22 | 5.30E-04 | 1.30E-02 |
