# Supplementary material for: Genome-wide scan for signatures of selection in Hanwoo and Angus cattle using whole-genome sequence data
Source: PLoS One. 2025 May 27;20(5):e0324034. doi: 10.1371/journal.pone.0324034 (PMC12111605; doi:10.1371/journal.pone.0324034)
Supplement: S3 Table — (DOCX) [file pone.0324034.s004.docx]

Table 1. The Gene Ontology (GO) enrichment analysis among overlapped genes from all the methods.

| Method | Breed | GO term | Gene symbol |
| --- | --- | --- | --- |
| iHS | Hanwoo | - Olfactory receptor activity  - G-protein coupled receptor activity  - Olfactory transduction  - Odorant binding  - Sensory perception of smell | *OR5AL8, OR5D77, OR5D79, OR5I1, OR5L1C, OR5L20, OR5W1, OR5W26, OR8A1, OR8A1C, OR8A1D, OR8A1E, OR8A1F, OR8B1, OR8B12, OR8B1AE, OR8B1AI, OR8B1AU, OR8B1R, OR8B1S, OR8B1T, OR8B3, OR8B4, OR8B69, OR8B8, OR8C26, OR8C7, OR8D2, OR8D6, OR8G3, OR8G3B, OR8G3F, OR8G3L, OR8G4, OR8G47, OR8G5, OR8G5B, OR8H13, OR8J16, OR8J19, OR8J3F, OR8K1, OR8K3B, OR8K5B, OR8K62, OR8K64, OR8K66* |
|  |  | - Prolactin receptor binding  - Positive regulation of lactation  - Response to nutrient levels  - Mammary gland development | *PRP3, PRP2, CSH2, PRP1, PRP-VII, PRL, PRP14, PRP6, PRP9, PRP8* |
|  | Angus | - Aspartic-type endopeptidase activity  - Protein digestion and absorption | *SLC6A19, MGC157405, PAG7, PAG6, PAG9, PAG21, PAG20, PAG11, PAG3, PAG17, PAG4, PAG19, PAG14, PAG1, MGC157408, PAG16, PAG15* |
| Rsb | Between-breeds | - Olfactory receptor activity  - G-protein coupled receptor activity  - Olfactory transduction  - Odorant binding | *OR12D23, OR14J1, OR12D2E, OR12D2D, OR12D2H, OR12D2G, OR12D2F, OR14J12, OR11W1, OR12D3, OR12D2, OR12D18* |
|  |  | - Hemoglobin alpha binding  - Haptoglobin-hemoglobin complex  - Organic acid binding  - Hemoglobin complex  - Oxygen transport  - Oxygen transporter activity  - Hydrogen peroxide catabolic process  - Oxygen binding | *HBE2, HBE4, HBB, HBG* |
|  |  | - Carbonate dehydratase activity  - Hydro-lyase activity  - Nitrogen metabolism | *CA1, CA2, CA3, CA6, CA13* |
| XP-EHH | Between-breeds | - Olfactory receptor activity  - G-protein coupled receptor activity | *OR51F2, OR51F1, OR51H1, OR51H8, OR51F5, OR52E2, OR51F4, OR51M1, OR52A1F, OR51K2, OR52A1C, OR51V1, OR52R1, OR52S2, OR51V1D, OR51S1, OR51A8B, OR52A1, OR52A5, OR51A24* |
|  |  | - Hemoglobin alpha binding  - Haptoglobin-hemoglobin complex  - Organic acid binding  - Hemoglobin complex  - Oxygen transport  - Oxygen transporter activity  - Hydrogen peroxide catabolic process  - Oxygen binding | *HBE2, HBE4, HBB, HBG* |
|  |  | - Alpha-(1,2)-fucosyltransferase activity  - Galactoside 2-alpha-L-fucosyltransferase activity | *FUT1, FUT2, SEC1* |
